# Rectal screening for carbapenemase-producing Enterobacteriaceae in an intensive care unit in India

**DOI:** 10.4314/gmj.v58i2.7

**Published:** 2024-06

**Authors:** Arun Sachu, Sanjo Sunny, Philip Mathew, Ajeesh Kumar, Alice David

**Affiliations:** 1 Department of Microbiology, Believers Church Medical College Hospital, Thiruvalla, Kerala, India; 2 Department of Critical Care, Believers Church Medical College, Thiruvalla, Kerala, India; 3 Medical Research, Believers Church Medical College Hospital, Thiruvalla, Kerala, India

**Keywords:** Carbapenem, Bacterial colonisation, Infection, Screening, Resistance

## Abstract

**Objectives:**

To determine the proportion of patients admitted to ICU who are colonised with carbapenem-resistant Enterobacteriaceae (CRE) and to estimate the agreement between colonised patients and patients who developed an infection with CRE.

**Design:**

Prospective surveillance study

**Setting:**

The ICU of a tertiary care hospital in Kerala, India

**Participants:**

All patients above 18 were admitted to the ICU during the study period.

**Outcome measures:**

Patients colonised with CRE and systemic infection with the colonised organism

**Results:**

CRE colonisation was found in 20(8.7%) samples. Among the 20 patients in the study who were colonised with CRE, 5(25%) developed systemic infection due to CRE. History of antibiotic usage and admission to other hospitals in the last 90 days were independent predictors of CRE colonisation.

**Conclusion:**

Five of the 20 patients colonised with CRE developed an infection. Hospital admission and antibiotic usage were the main risk factors associated with CRE colonisation. Antibiotic escalation was suggested for two colonised patients based on their clinical worsening, but they succumbed to the illness. This study led us to modify our infection control practices, which led to isolating patients colonised with CRE.

**Funding:**

None declared

## Introduction

Patients in Intensive care Units (ICUs) are susceptible to nosocomial infections due to prior antibiotic use, underlying diseases and use of multiple invasive procedures and devices such as indwelling catheters that increase the risk of infections.^1^,^2^ Patients in the ICU are also at risk for colonisation and infection with Multi Drug Resistant Organisms(MDROs). These organisms include Methicillin-resistant *Staphylococcus aureus*(MRSA), Vancomy cin-resistant *enterococci* (VRE), Carbapenem-resistant *Acinetobacter baumannii* (CRAB), organisms that produce extended-spectrum β-lactamases (ESBL) and Carbapenem-resistant Enterobacteriaceae(CRE).^3^,^4^ CRE has become a threat for ICU patients because of limited treatment options and increased mortality.^5^,^6^ The widespread use of Carbapenems has made infection/colonisation with CRE an important challenge in high-risk patients. CRE was first identified in 2001 and has been disseminated widely since then.^7^ CRE refers to bacteria belonging to the Enterobacteriaceae family that can survive and grow in clinically relevant concentrations of carbapenems.^8^ The Centers for Disease Control and Prevention (CDC) defines CRE as bacteria that are non-susceptible to any carbapenem or are documented to produce carbapenemases.^9^ CRE can be divided into two main groups: Carbapenemase-producing (CP-CRE) and Non-carbapenemase-producing CRE. In settings with high CRE prevalence, carbapenemase-producing Enterobacteriaceae (CPE) usually contributes to most CRE isolates from clinical sources.^10^ Most but not all CRE are CPE and vice versa. Some CRE are not CPE (i.e., those with a carbapenem resistance mechanism other than carbapenemase production). Some CPEs are not CRE (i.e., those which exhibit low carbapenem MICs and remain phenotypically susceptible to carbapenems). Other Carbapenem resistance mechanisms include AmpC beta-lactamases, ESBLs, Porin mutations and Efflux pumps.^11^,^12^

CP-CRE produce many carbapenemases, which can be divided into three main groups according to the Ambler classification: class A, B and D beta-lactamases.^13^,^14^ The major Carbapenemase within Class A is the clinically relevant *Klebsiella pneumoniae* carbapenemase. (KPC).^15^ It is partially inhibited by clavulanic acid and KPC has spread worldwide and has been found in clinical isolates of *Klebsiella, E.coli, Salmonella, Citrobacter freundii, Enterobacter aerogenes, Enterobacter cloacae, Proteus mirabilis* and *Serratia marcescens*.^16^,^17^,^18^,^19^,^20^ Another major carbapenemase family belonging to class B are MBLs(Metallo Beta lactamases). These enzymes depend on the interaction with zinc ions in the enzyme's active site.^21^ They include the New Delhi metallo-beta-lactamase 1 (NDM-1), Imipenem-resistant *Pseudomonas* (IMP)-type carbapenemases, and the Verona integron-encoded metallo-beta-lactamases (VIM).^22^ NDM-1, IMP and VIM were first detected in India, Japan and Italy.^23^,^24^,^25^ The third clinically relevant group of carbapenemases are OXA-48-like, which belongs to Ambler class D. Six OXA-48-like variants have been identified, of which OXA-48 is the most widespread. They are commonly found in *K. pneumoniae, E. coli, C. freundii* and *E. cloacae*.^26^,^27^

Colonisation by carbapenem-resistant Enterobacteriaceae (CRE) is an important cause of infection and one of the main sources of CRE dissemination in hospitals and communities.^28^ In asymptomatic carriers, the main CRE reservoir is the microbiota in the gastrointestinal tract, followed by the oropharynx, skin and urine.^29^ Active CRE surveillance testing is an important strategy to control the spread of CRE, as it allows early implementation of contact isolation, resulting in better patient care.^30^ CDC considers the detection of CRE through rectal swabs as the preferable method of CRE screening. 31 The prevalence of CRE colonisation in hospitalised patients ranges from 3% to 7%, but it can be higher in patients admitted to critical care units (CCUs). In one Indian study, it was found that the prevalence of CRE in CCU ranged between 13% and 51%.^32^,^33^,^34^ Patients with CRE colonisation have a high probability of developing a subsequent infection that may be associated with bacteremia, leading to increased morbidity and mortality.^33^

Common risk factors for CRE acquisition in hospitals include exposure to antimicrobials, co-morbidities, recent stay in a long-term care facility(LTCF), history of recent invasive procedures or permanent foreign devices, and recent hospital admission.^35^,^36^ In some countries, like Israeli LTCFs, screening is recommended for all new admissions that are transferred directly from an acute-care hospital or patients admitted from home with extensive healthcare exposure.^37^ Both the CDC and European Society of Clinical Microbiology and Infectious Diseases (ESCMID) support additional periodic screening policies in the facility (e.g., weekly) for hospitalised patients in high-risk units.^5^ In India, no such screening policy is uniformly followed. As with any screening program, decisions about which patients to screen should be based on local epidemiologic data.^5^ Studies have shown that CRE colonisation is higher among hospitalised patients in the ICU when compared to other areas.^33^ Olivgeris et al. showed that 61.5% of the patients colonised with CRE had a history of recent ICU admission, while only 1.7% of the non-colonizers had a history of ICU admission.^33^ Asymptomatic carriage of CRE increased the risk of infection by 8.8% and 27% among hospitalised and ICU patients, respectively.^32^,^38^ Patients with CRE infections usually have a history of long-term exposure to healthcare facilities due to unrelated co-morbidity. This exposure can result in long-term and varied antibiotic use, leading to the gradual development of severe and more resistant bacterial infections like CRE. CRE genes do not confer increased pathogenicity, making initial presentation similar to other infections caused by a less resistant strain of the same organism. The key indicator of CRE infections is the uncontrolled progression of the illness, leading to a very severe disease state despite empiric antibiotic intervention.^39^

The aim of the study was to

1. To determine the proportion of patients admitted to our ICU who are colonised with CRE.
2. To estimate the agreement between colonised patients and patients who developed an infection with CRE.
3. To enumerate risk factors associated with colonisation.
4. To assess the effect of early detection of colonisation in terms of choice of antibiotics
5. To guide the implementation of interventions to prevent the transmission of CRE.

## Methods

This prospective surveillance study was done in an ICU of a tertiary care hospital between November 2021 and August 2022 after getting clearance from the Institutional Ethical Committee (Reg No: ECR/1098/Inst/KL/2018). Informed consent was taken from patients.

Two rectal swabs were collected from all patients more than 18 years of age who were admitted to the ICU as part of the Hospital Protocol. Patients were assessed using a detailed questionnaire which included duration of stay in other hospitals, exposure to high-end antibiotics (carbapenem, colistin, tigecycline, polymyxin B, vancomycin, teicoplanin), devices inserted, history of any comorbid conditions like diabetes mellitus, chronic kidney disease, surgery done in the past 90 days and past history of colonisation and infection with CRE.

The culture media used in the study was chromID® CARBA SMART Agar (bioMerieux, Marcy L'Etoile, France), which consisted of two chromogenic culture media dispensed into one Petri dish containing separate compartments (CARB/OXA). Media contains a mixture of antibiotics, which enables the selective growth of mainly KPC and metallo-carbapenemase-type CPE for the CARB medium and OXA-48-type CPE for the OXA medium.

**Figure 1:**
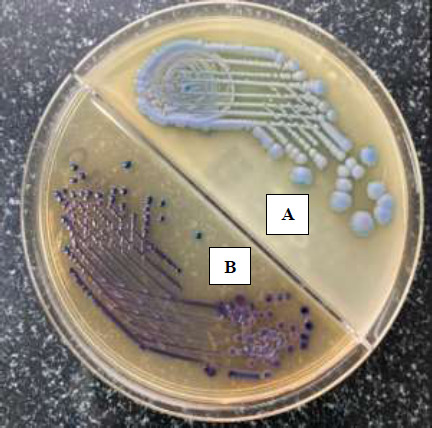
Growth pattern in CARB and OXA portion of CHROM ID CARBA SMART Agar. **A.** Bluish- to bluish-grey colonies suggestive of *Klebsiella, Enterobacter, Serratia, Citrobacter* (KESC) growing on the CARB portion **B.** Dark Pink to Burgundy colour colonies suggestive of Escherichia coli growing on the OXA portion

One rectal swab from each patient was inoculated into the CARB portion of the medium and the other into the OXA portion. The plates were incubated at 37°C for 24 hrs. The growth seen in plates is shown in Figure 1. Each colony was subcultured onto MacCkonkey agar, and identification and carbapenem resistance was confirmed by VI-TEK-2 (bioMe′rieux, Marcy l'Etoile, France).

### Statistical Analysis

Descriptive statistics included proportions for all variables except age, summarised as mean and standard deviation. The difference in infection rate was tested using a test of proportions. The Chi-square Test was used to test association. Odds Ratio with 95% Confidence Interval was used to estimate the risk of colonisation causing infection and the odds of potential risk factors of CPE colonisation. Logistic Regression was used to identify independent predictors of CPE colonisation

## Results

Rectal swabs (458) were collected from 229 patients, of whom 102 were transferred from other Health Care Facilities (HCF), and 127 were directly admitted from the community. The mean age of the study patients was 64.0 + 17.3 years; 164(71.6%) were males, and 65(28.4%) were females.

CRE colonisation was found in 20(8.7%) samples. Of the 102 patients from other HCF,12 (11.8%) patients had rectal colonisation with CRE. Of the 127 patients from the community,8 (6.3%) had CRE colonisation. Among the 8 patients received from the community who were found to have CRE colonisation,5 patients had a history of admission in another hospital in the last 3 months. Among the 20 isolates of CRE colonised, one isolate of Carbapenem-resistant Klebsiella produced growth both in the CARB and OXA portion of the plate, indicating the presence of metallo-beta-lactamase/KPC and OXA-48 type resistance mechanism. The remaining 19 isolates produced growth only in the CARB portion, indicating the presence of a Metallo-beta-lactamase/KPC-type resistance mechanism.

Among the 20 colonisers,17(85%) patients were colonised with Carbapenem-Resistant Klebsiella, while the remaining 3(15%) patients were colonised with Carbapenem-Resistant E. coli. Among the 20 patients in the study who were colonised with CRE,5(25%) developed systemic infection due to CRE. Among the 209 patients who were not colonised with CRE, only 7(3.3%) developed systemic infection due to CRE. The difference in infection rate was highly significant (p<0.00001), and the odds of a patient with colonisation having a subsequent infection was almost as high as 10:1 (OR (95%CI): 9.6 (2.7-34.0)). Two of the 5 CRE colonisers who developed systemic infection had urinary tract infections due to carbapenem-resistant *Klebsiella*(CRK). The remaining 3 patients had urinary tract infections caused by CRK and Carbapenem-resistant E. coli, pneumonia due to CRK and urosepsis due to CRK, respectively. None of the patients received from the community who were colonised developed any systemic infection due to CRE. Among the 20 patients colonised with CRE, antibiotics were escalated for only two patients based on their colonisation status. Both these patients developed an infection with CRE and unfortunately succumbed to the illness.

As shown in Table 1, several potential risk factors such as history of hospital admission in the last 90 days, history of surgery in the last 90 days, history of intubation, Central Line, Foleys Catheter and Antibiotic usage in the last 90 days were significantly associated with colonisation of CRE. The only comorbid condition that is a potential risk factor is chronic liver disease. After multivariate analysis, only hospital admission in the last 90 days[Odds Ratio (OR) 3.5, 95% Confidence Interval (CI) 1.1–11.1, p-value-0.03] and Antibiotic usage in the last 90 days[Odds Ratio (OR) 4.7, 95% Confidence Interval (CI) 1.6–13.6, Pvalue-0.004] were independent predictors of colonisation with CRE (Table 2).

**Table 1:** Potential risk factors associated with CPE colonisation

Potential Risk Factors	Total (n=229)	CPE Colonization		p Value
		Yes (n=20)	No (n=209)	
**Male**	164 (71.6%)	13 (65.0%)	151 (72.3%)	0.6
**Patient from other HCF**	102 (44.5%)	12 (60.0%)	90 (43.1%)	0.2
**History in the Last 90 Days**				
Hospital Admission*	82 (35.8%)	15 (75.0%)	67 (32.1%)	0.0003
Surgery*	18 (7.9%)	4 (20.0%)	14 (6.7%)	0.05
Intubation*	24 (10.5%)	5 (25.0%)	19 (9.1%)	0.04
Central Line*	8 (3.5%)	3 (15.0%)	5 (2.4%)	0.02
Foley Catheter*	78 (34.1%)	13 (65.0%)	65 (31.1%)	0.005
**Tracheostomy**	7 (3.1%)	2 (10.0%)	5 (2.4%)	0.1
**Any Microorganism Isolated**	5 (2.2%)	1 (5.0%)	4 (1.9%)	0.4
Antibiotic Use*	45 (19.7%)	12 (60.0%)	33 (15.8%)	<0.0001
**Antifungal Use**	1 (0.4%)	1 (5.0%)	0 (0.0%)	0.09
**Comorbid Conditions**				
**Diabetes Mellitus**	118 (51.5%)	14 (70.0%)	104 (49.8%)	0.1
**Neurological Disease**	71 (31.0%)	7 (35.0%)	64 (30.6%)	0.8
**Dementia**	14 (6.1%)	2 (10.0%)	12 (5.7%)	0.4
**Myocardial Infarction**	22 (9.6%)	3 (15.0%)	19 (9.1%)	0.4
**Congestive Heart Failure**	20 (8.7%)	3 (15.0%)	17 (8.1%)	0.4
**Peripheral Vascular Disease**	12 (5.2%)	1 (5.0%)	11 (5.3%)	1
**Cardiovascular Accident**	57 (24.9%)	3 (15.0%)	54 (25.8%)	0.4
**Chronic Kidney Disease**	29 (12.7%)	3 (15.0%)	26 (12.4%)	0.7
Chronic Liver Disease*	35 (15.3%)	7 (35.0%)	28 (13.4%)	0.02

* Significant at p-value <0.05, HCF- Health Care Facility

**Table 2:** Independent risk factors associated with CPE colonisation

Independent Risk Factors	Total (n=229)	Risk of CPE Colonization		
		OR (95% CI	Adjusted OR (95% CI	p Value
**Male**	164 (71.6%)	0.7 (0.3-1.9)		
**Patient from other HCF**	102 (44.5%)	2.0 (0.8-5.1)		
**History in the Last 90 Days**				
**Hospital Admission**	82 (35.8%)	6.4 (2.2-18.2)*	3.5 (1.1-11.1)	0.04
**Surgery**	18 (7.9%)	3.5 (1.03-11.8)*	NS	
**Intubation**	24 (10.5%)	3.3 (1.1-10.2)*	NS	
**Central Line**	8 (3.5%)	7.2 (1.6-32.7)*	NS	
**Foley Catheter**	78 (34.1%)	4.2 (1.6-10.8)*	NS	
**Tracheostomy**	7 (3.1%)	4.5 (0.8-25.0)	.	
**Microorganism**	5 (2.2%)	2.7 (0.3-25.4)	.	
**Antibiotic Use**	45 (19.7%)	8.0 (3.0-21.1)*	4.7 (1.6-13.6)	0.004
**Antifungal Use**	1 (0.4%)	N/A		
**Comorbid Conditions**				
**Diabetes Mellitus**	118 (51.5%)	2.4 (0.9-6.4)		
**Neurological Disease**	71 (31.0%)	1.2 (0.5-3.2)		
**Dementia**	14 (6.1%)	1.8 (0.4-8.8)		
**Myocardial Infarction**	22 (9.6%)	1.8 (0.5-6.6)		
**Congestive Heart Failure**	20 (8.7%)	2.0 (0.5-7.5)		
**Peripheral Vascular Disease**	12 (5.2%)	0.9 (0.1-7.7)		
**Cardiovascular Accident**	57 (24.9%)	0.5 (0.1-1.8)		
**Chronic Kidney Disease**	29 (12.7%)	1.2 (0.3-4.5)		
**Chronic Liver Disease**	35 (15.3%)	3.5 (1.3-9.5)*	NS	

* Significant at p< 0.05, NS: Not Significant, N/A- Not Applicable, HCF- Health Care Facility

## Discussion

Patients colonised by Multidrug-resistant bacteria are considered to be important reservoirs since they favour horizontal transmission of these microorganisms in the hospital environment.^40^ CRE carry genes that often confer high-level resistance to Beta-lactams, Beta-lactam/beta-lactamase inhibitors, and Carbapenems, often leading to limited therapeutic options. The rapid dissemination of CRE worldwide is a cause for grave concern and has become a global health crisis. So controlling their spread is of utmost importance.^41^ WHO included CRE as a critical pathogen in the priority list published in 2017.^42^

CRE colonisation can be detected by different culturebased methods, as shown in Table no 3.^43^,^44^. A comparison of several molecular methods for CRE detection showed that Xpert Carba-R, Eazyplex Superbug complete A and Check-Direct CPE kit demonstrated 100% sensitivity for KPC, NDM and VIM. Still, only the CHECK-Direct CPE detected all OXA-48 genes.^45^ Molecular methods for CRE detection are very costly and unavailable in most laboratories. Chromogenic medium chromID® CARBA SMART Agar(bioMerieux, Marcy L'Etoile, France) was used in our study since chromogenic media have advantages such as ease of detection, shorter turnaround time and high sensitivity.^43^

**Table 3:** Culture-based screening methods for CRE

Methods	Sensitivity(%)	Specificity(%)
**CDC protocol**	98.8	80.2
**MA with Carbapenem**	75.8	89.6
**MA with Meropenem**	89.1	85.2
**Chrom ID Carba**	96.5	91.2
**Super Carba**	80	98.5
**Spectra CRE**	97.8	86.4
**Agar Dilution with Carbapenem**	84.9	94.3

Our study found CRE colonisation in 20(8.7%) samples. Another study by Ramanathan et al. in India reported a CRE prevalence of 7.8% in ICU.^46^ CRE colonisation rates in different studies are shown in Table 4. In this study, 11.8% of patients received from other healthcare facilities and 6.3% of the patients received from the community were found to be colonised with CRE. This was similar to the findings in the study conducted by Ramanathan et al., which showed that CRE colonisers were more common among patients received from other healthcare facilities.^46^ Studies conducted by Ramanathan et al., Garpvall et al., and Gomides et al. showed that CRE infections developed in 37.5%,14% and 20.54% of the individuals colonised with CRE.^46^,^50^,^51^ Our study was concordant with these findings and showed that CRE infection developed among 25% of the individuals colonised with CRE. This was statistically significant as compared to non-colonizers. Among the five patients colonised with CRE who developed an infection with CRE, the colonising and infecting organisms were the same in four patients. One patient colonised with Carbapenem-Resistant Klebsiella developed an Urinary Tract infection with both Carbapenem-resistant Klebsiella and Carbapenem-Resistant *E.coli*.

**Table 4:** CRE colonisation in different studies

Author	Place of Study	CRE colonization(%)	Method of Testing
**Ramanathan et al^46^**	India	7.8	Agar dilution with 1 µg/ml of Carbapenem
**McConville et al^47^**	Manhattan, America	28	VITEK-2 Susceptibility
**Banach et al^48^**	New York,America	2.6	Modified Hodge Test
**Shimasaki et al^49^**	Chicago, America	3.3	Multiplex PCR
**Garpvall et al^50^**	Vietnam	35.8	Chrom ID Carba
**Gomides et al^51^**	Brazil	15.47	VITEK-2 Susceptibility
**This study**	India	8.7	Chrom ID Carba Smart

The risk factors analysed were patients received from other health care facilities and community, History of (h/o) hospital admission in the last 90 days, h/o surgery in the last 90 days, Intubation, Central Line, Diabetes Mellitus, Chronic Kidney Disease, Foleys Catheter, Chronic Liver Disease and h/o Antibiotic usage in the last 90 days. Hospital admission in the last 90 days and h/o Antibiotic usage in the last 90 days were independent risk factors for colonisation with CRE. A study conducted by Ramanathan et al. showed that h/o antibiotic usage and h/o surgery in the last 90 days were statistically associated with CRE colonisation, which was concordant with the findings in our study.^46^ Antibiotic usage as a risk factor for CRE colonisation was also mentioned by Gomides et al. and Swaminathan et al.^51^,^52^ The majority of CRE infections worldwide are caused by *K. pneumoniae* as reported by Tzouvelekis LS et al.^53^ Our study showed that 85% of the colonisers were due to Carbapenem-Resistant *Klebsiella* and majority of the infections were due to the same organism. Gomides et al. showed a similar finding to our study in that 83.16% of the CRE colonisers were due to *Klebsiella pneumoniae*.^51^ A study conducted in Vietnam by Garpvall et al. showed that *Escherichia coli* was the major(41%) CRE coloniser, which was discordant to the findings in our study.^50^

Chromogenic agars used for detecting CRE colonisation can shorten the turnaround time but are not as sensitive as molecular methods, which are costly.

Examples include Brilliance CRE (Thermo Diagnostics, USA), CHROMagar KPC (CHROMagar, France), chromID Carba (bioMerieux, France), chromIDOXA-48 (bioMerieux, France) and Supercarba (bioMerieux, France).^43^ Viau et al. suggested that chromID Carba should be used for culture-based screening unless OXA-48 incidence is high, which then merits the addition of an OXA-48-specific medium (e.g., chromID OXA-48) or Supercarba.^53^ In our study, Chrom IDCARBA SMART Agar proved to be an excellent medium as it can detect KPC, Metallobetalactamse and OXA-48 simultaneously. In this study, only one isolate showed an OXA-48 type resistance mechanism, which might be because CHROM ID medium has reduced sensitivity for detection of this resistance mechanism.^53^

Among the 20 patients colonised with CRE, antibiotic escalation was suggested for two patients on clinical worsening based on their colonisation status. Antibiotics were escalated to Ceftazidime avibactam, but both patients developed an infection with CRE and succumbed to the illness. The low colonisation rate of CRE, coupled with the association of antibiotic usage and admission in other hospitals as independent risk factors for CRE colonisation, guided us to propose risk factor-based active surveillance. Our infection control policy based on isolating patients with CRE infection was modified to include the colonisers. This led to pre-emptive contact isolation of colonisers, proper staff allocation, dedicated equipment and environmental cleaning. These policy changes will hopefully lead to decreased spread of CRE in the hospital. Antibiotic usage as a risk factor further stresses the need for Antibiotic Stewardship. Antimicrobial stewardship optimises antimicrobial use in terms of selection, dosing, and duration while minimising toxicity. Consequently, judicious antimicrobial use would be expected to reduce the selective pressure favouring these highly resistant pathogens.^54^ Control of CRE infection is an important aspect of antimicrobial stewardship. Active surveillance with rectal cultures and the early initiation of infection control procedures resulted in a 4.7-fold reduction in CRE infection in an outbreak setting at an Israeli hospital.^55^ Antimicrobial Stewardship program was already initiated in our hospital before this study.

### Limitations

Our study had a few limitations. Rectal screening for CRE was done only at admission and was not repeated thereafter. Repeat swabs were not taken in colonised patients to look for de-colonisation. Molecular methods could not be used for CRE screening due to financial limitations.

Although Chrom IDCARBA SMART Agar could find the resistance mechanism, the media cannot differentiate between KPC and Metallo-beta-lactamase. The reduced detection of the OXA-48 resistance mechanism might be due to the medium's sensitivity.

## Conclusion

Overall, the CRE colonisation rates among patients admitted to our hospital's ICU were quite low(8.7%). Among the 20 patients in the study who were colonised with CRE, 5(25%) developed systemic infection due to CRE. History of antibiotic usage and admission to other hospitals in the last 90 days were independent predictors of CRE colonisation. Antibiotic escalation was suggested for two colonised patients based on their clinical worsening, but they succumbed to the illness. Hence, rather than screening all patients admitted to the ICU, which will not be cost-effective, an active risk factor-based surveillance strategy is proposed, resulting in better utilisation of resources.
